# 

**Published:** 1898-12-17

**Authors:** 


					j. iic aiam
fluid HA
CADBURY-S rf H ? CAD<SH?YS
ABSOLUTELY PURE. NO DRUGS OR ALKALIES USED.
- ' ? g - ~?-fiQMQUt^HORW!CH HOST.UAL
wo. 638. Vol. XXV. Saturday,
Deo. 17th, 1898.
L .mp.192. J PRICE TWOPENCE.

				

